# Irritable Bladder from Tape-worm

**Published:** 1848-10

**Authors:** 


*Irritable Bladder from Tape-worm.* By Mr. Tufnell.—A man of temperate habits complained of excessive irritability of the bladder, with difficult micturition. His health has been good till three months previously, when he began to suffer from dyspepsia, with irritation of the rectum and hemorrhoids. These symptoms increased, and to them were added tenesmus, and frequent calls to make water, which was voided in a twisted jet, and accompanied by severe straining, but no pain. The urine was highly acid, and loaded with lithate of ammonia. The prostrate was of natural size, but very sensitive to the touch. His symptoms were twice removed by appropriate treatment, but he returned a third time, suffering severely, and anxiously desiring an operation for his relief, being convinced that he suffered from urinary calculus. The irritation about the anus had now greatly increased, and he was observed at the same time to be frequently rubbing his nose, which led to the suspicion of worms in the intestines. A purgative of turpentine and castor-oil was administered, and the following morning a tape-worm, measuring thirty feet, was evacuated. The patient obtained immediate relief from his distressing symptoms.—*Dublin Medical Press.*

